# Sodium–Glucose Cotransporter 2 Inhibitors Shorten Echocardiography-Derived Total Atrial Conduction Time in Patients With Type 2 Diabetes Mellitus: A Prospective Pilot Study

**DOI:** 10.31083/RCM50764

**Published:** 2026-05-26

**Authors:** Ercan Taştan, Fethullah Kayan, Songül Beskisiz

**Affiliations:** ^1^Department of Cardiology, Diyarbakır Selahaddin Eyyubi State Hospital, 21100 Diyarbakır, Turkey; ^2^Department of Cardiology, SBU Diyarbakır Gazi Yaşargil Training and Research Hospital, 21070 Diyarbakır, Turkey; ^3^Department of İnternal Medicine, Diyarbakır Gazi Yaşargil Training and Research Hospital, 21070 Diyarbakır, Turkey

**Keywords:** sodium-glucose cotransporter 2 inhibitors, type 2 diabetes mellitus, atrial remodeling, echocardiography, cardiac conduction, diastolic dysfunction

## Abstract

**Background:**

Sodium-glucose cotransporter 2 inhibitors (SGLT2i) have been shown to improve cardiovascular outcomes in patients with type 2 diabetes mellitus (T2DM). Structural and electrical atrial remodeling are among the myocardial alterations associated with diabetes. Total atrial conduction time (TACT), derived from echocardiography, serves as a marker of atrial remodeling. However, the effect of SGLT2i on TACT remains unclear.

**Methods:**

In this prospective, single-center pilot study, 130 patients with T2DM and preserved left ventricular ejection fraction were enrolled between March and December 2022. After excluding patients who discontinued therapy or met other exclusion criteria, 107 patients (57 treated with dapagliflozin and 50 with empagliflozin) were included in the analysis. Echocardiographic and laboratory evaluations were performed at baseline and six months after initiation of SGLT2i therapy. TACT was defined as the mean time between the onset of the P wave in lead II and the peak A′ wave on tissue Doppler imaging (PA-TDI). Paired statistical tests, correlation analyses, and multiple linear regression were used to identify independent predictors of Δ TACT.

**Results:**

After six months of SGLT2i therapy, significant reductions were observed in blood pressure, lipid levels, glycated hemoglobin (HbA1c), and body mass index values (all *p* < 0.05). Moreover, echocardiography demonstrated significant decreases in the E/e′ ratios and mean PA-TDI duration (*p* < 0.001). The Δ lateral E/e′ ratio and Δ body mass index (BMI) values were independently associated with Δ TACT (*p* = 0.001 and *p* = 0.026, respectively). The mean duration of SGLT2i use was 183 days.

**Conclusions:**

SGLT2i therapy was associated with a significant reduction in TACT among T2DM patients, suggesting potential improvements in atrial remodeling and diastolic function. These findings support the hypothesis that the cardiovascular benefits of SGLT2i may extend to atrial conduction properties. Nonetheless, larger randomized studies are warranted to confirm these observations.

## 1. Introduction

Type 2 diabetes mellitus (T2DM) is an important cardiovascular risk factor, and its global prevalence is estimated to reach 600 million by the year 2045 [1]. Sodium-glucose cotransporter 2 (SGLT2) inhibitors constitute a new class of glucose-lowering agents. A large number of clinical trials have demonstrated that SGLT2 inhibitors improve the cardiovascular outcomes of T2DM patients [2, 3, 4, 5]. Structural changes (increased fibrosis and hypertrophy) and electrical remodeling (increased Ca^2+^ handling abnormalities) of the atria are among the myocardial changes induced by diabetes mellitus (DM) [6, 7]. Studies have shown that SGLT2 inhibitors reduce oxidative stress and improve mitochondrial function, thereby reducing atrial fibrosis and hypertrophy [8, 9, 10].

Echocardiographic assessment of total atrial conduction time (TACT) is a marker of the morphological (atrial size), electrical (ion channel alterations) and structural changes (e.g., fibrosis and hypertrophy) associated with atrial remodeling [11]. TACT increases in the presence of valvular disease, hypertension, a high body mass index (BMI), a history of atrial fibrillation (AF) and a large left atrial diameter. An increase in TACT is a predictor of AF [11, 12]. Additionally, TACT can be used to predict AF recurrence after radiofrequency catheter ablation (RFCA) [13]. Prolonged TACT in heart failure predicts poor cardiac prognosis (including mortality) [14]. It should be noted that there is limited evidence in the literature that TACT prolongation is reversible [15].

Considering the above, we aimed to explore whether SGLT2 inhibitors may reduce TACT in patients with T2DM, as a hypothesis-generating approach.

## 2. Methods

### 2.1 Study Population

This prospective, single-center pilot study was performed to evaluate the effects of additional treatment with SGLT2 inhibitors on the TACTs of T2DM patients. Between March 2022 and December 2022, 130 T2DM outpatients who had inadequately controlled were consecutively recruited from Gazi Yaşargil Training and Research Hospital, Diyarbakir, Turkey. All participants were evaluated at baseline and six months after commencing treatment with SGLT2 inhibitors. The 130 patients recruited had normal ejection fractions (EFs) and normotension or regulated hypertension.

The inclusion criteria were as follows: (1) age >18 years, (2) normotension or regulated hypertension, (3) ejection fraction ≥50%, (4) normal sinus rhythm and without a left bundle branch block or right bundle branch block. The following were the exclusion criteria: (1) previous valve operation or a history of severe valvular disease; (2) low GFR (glomerular filtration rate <50 mL/min with the Modification of Diet in Renal Disease (MDRD) formula); (3) initiation or dose modification of antihypertensive, lipid-lowering, beta-blocking, or diuretic agents during the six-month period after the start of this study; (4) presence of acute coronary syndrome or any other cardiac disease with positive troponin (such as myocarditis, myocardial infarction with nonobstructive coronary arteries (MINOCA)), percutaneous coronary intervention (PCI) or coronary bypass during the six-month period after the start of this study; (5) P waves with different morphologies on electrocardiograms (ECGs). During the follow-up period, we excluded six patients from the study because of additional drug use (antihypertensives, statins and fenofibrate), two patients because of a history of PCI and new AF, four patients for discontinuing the SGLT2 inhibitors, and 11 patients did not show up for the follow-up measurements six months after starting treatment. We excluded a total of 23 (6+2+4+11) patients from the study during the follow-up period. The final study sample consisted of 107 patients. Among them, 57 patients were on dapagliflozin, and 50 were on empagliflozin. Dapagliflozin and empagliflozin were analyzed together as they would both have similar class effects, despite possible pharmacological differences.

The absence of a control group introduces potential confounding bias. But as a prospective within-subject design, our study evaluated pre- and post-treatment parameters in the same individuals, which partially minimizes inter-individual variability. Nevertheless, we have now clarified this limitation in the Discussion section. Furthermore, clinically relevant covariates, particularly age, sex, Δ BMI, and Δ lateral E/e^′^ ratio, were evaluated in the regression analysis. Because of the sample size and the risk of overfitting, not all potential confounders were entered into the final multivariable model.

The study protocol conformed to the Declaration of Helsinki and was approved by the ethics committee of Gazi Yaşargil Training and Research Hospital (approval number: 2022-50; date: March 11, 2022).

### 2.2 Echocardiographic Measurement

Vivid S70 (GE Healthcare, Horton, Norway) was used to obtain the echocardiographic images. Three consecutive heart cycles were recorded, and images were obtained at a frame rate of 60–80 frames per second. ECGs were taken while echocardiography was being performed. Although formal blinding was not implemented, echocardiographic analyses were performed independently by two experienced cardiologists, and the mean of their measurements was used for analysis. To assess diastolic function, the following mitral pulsed-wave Doppler and tissue Doppler parameters were measured: peak early (E) and late (A) diastolic filling velocities, E/A ratio, septal early diastolic mitral annular tissue velocity (septal e^′^) and lateral early diastolic mitral annular tissue velocity (lateral e^′^), and lateral E/e^′^ and septal E/e^′^ ratios. The left atrial (LA) maximum and minimum volumes were measured in four- and two-chamber views according to the biplane area-length method. The right atrial (RA) maximum and minimum volumes were measured in the four-chamber view at the ventricular end-systole.

**Fig. 1.**
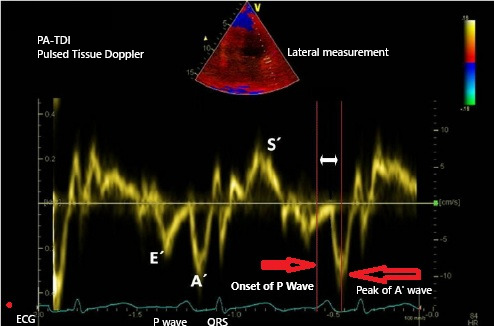
**PA-TDI interval: time from the onset of the P wave in lead II on ECG to the peak A^′^ wave on tissue Doppler imaging**. ECG, electrocardiogram; PA-TDI, the onset of the P wave in lead II and the peak A^′^ wave on tissue Doppler imaging.

TACT measurement was performed according to the recommendations of the American Society of Echocardiography (ASE) and the European Association of Cardiovascular Imaging (EACVI) [16]. The onset of the P wave was defined as the first deviation from the isoelectric line in lead II. TACT was calculated as the mean time across six different regions between the onset of the P wave in lead II of an ECG and the peak A^′^ wave obtained from tissue Doppler imaging (the onset of the P wave in lead II and the peak A^′^ wave on tissue Doppler imaging [PA-TDI] duration; Fig. 1). These values indicate high reproducibility of TACT measurements. The region of interest was just above the mitral annulus in the four-chamber view of the LA lateral and septal walls, above the tricuspid annulus in the four-chamber view of the RA free wall, in the two-chamber view of the LA posterior and anterior walls and in the three-chamber view of the LA inferolateral wall. PA-TDI was measured for the septal, lateral, posterior, anterior, inferolateral and RA free walls separately. The average of all measured intervals was accepted as the mean PA-TDI duration and the TACT equivalent. Although lateral PA-TDI is commonly used in clinical practice, we preferred averaging measurements from multiple atrial regions to better reflect global atrial conduction and reduce regional variability. This approach is supported by prior studies suggesting that multi-site assessment may provide a more comprehensive evaluation of atrial electromechanical properties [17, 18]. The intra- and interobserver coefficients of variation for the mean PA-TDI duration were 2.32% and 2.86%, respectively, at baseline and 2.56% and 3.12%, respectively, six months after treatment with SGLT2 inhibitors.

### 2.3 Statistical Analysis

Data analyses were performed using the Statistical Package for the Social Sciences 24.0 software (IBM, Armonk, NY, USA). Normality of distribution was assessed using the Kolmogorov–Smirnov test. Normally distributed variables were expressed as mean ± SD, while non-normally distributed variables were expressed as median (interquartile ranges [IQR]; 25–75%). The paired *t*-test or Wilcoxon signed-rank test was used to compare the variables at baseline and six months after treatment (Table 1). Pearson’s correlation or Spearman’s rank correlation coefficient was used to determine single correlations (Table 2). A multiple linear regression was used to assess the individual and cumulative effects of Δ BMI, Δ lateral E/e^′^ ratio, age and sex on the mean PA-TDI duration. Independent variables were selected from the variables that were significantly correlated with the mean PA-TDI duration (Table 3).

**Table 1.** **Comparison of variables between baseline and 6 months after treatment**.

	Pre-SGLT2i	Post-SGLT2i	*p*-value
BMI (kg/m^2^)	30.9 (27.4–34.6)	29.7 (26.1–32.8)	<0.001
SBP (mmHg)	125 (120–140)	125 (120–130)	<0.001
DBP (mmHg)	80 (76–80)	80 (75–80)	<0.001
Creatinine (mg/dL)	0.8 (0.65–0.90)	0.7 (0.60–0.85)	<0.001
eGFR (mL/min/1.73 m^2^)	90 (85–90)	90 (89–90)	0.261
Total cholesterol (mg/dL)	193 (170–225)	184 (167–215)	0.011
Triglycerides (mg/dL)	166 (114–236)	156 (105–218)	0.034
LDL cholesterol (mg/dL)	120 (97–141)	113 (95–132)	0.005
HDL cholesterol (mg/dL)	40 (34–49)	42 (36–49)	0.007
HbA1c (%)	8.3 (7.2–10.9)	7.1 (6.5–8.1)	<0.001
Hematocrit (%)	42 (40–45.4)	43.9 (41.6–46.2)	<0.001
PA-TDI (4Ch lateral) (ms)	129 (118–137)	118 (110–126)	<0.001
PA-TDI (4Ch septal) (ms)	115 (107–126)	103 (95–115)	<0.001
PA-TDI (2Ch anterior) (ms)	122 (110–133)	114 (103–124)	<0.001
PA-TDI (2Ch posterior) (ms)	110 (99–120)	99 (90–110)	<0.001
PA-TDI (3Ch posterolateral) (ms)	126 (118–137)	118 (107–126)	<0.001
PA-TDI RA (ms)	120 (110–129)	110 (99–122)	<0.001
Mean PA-TDI (ms)	121.1 (111.6–129.8)	110.5 (101.1–119.6)	<0.001
Lateral E/e′ ratio	7 (5.8–8.8)	6 (5.0–7.5)	<0.001
Septal E/e′ ratio	8.3 (7.1–10)	7.5 (6.2–8.8)	<0.001
E/A ratio	0.77 (0.60–0.93)	0.86 (0.70–1.25)	<0.001
LA max volume (mL)	57.5 (52.3–61)	58.1 (52.8–60.9)	0.159
LA min volume (mL)	36.9 (33.2–40.4)	37.5 (33.1–40.7)	0.8
RA max volume (mL)	54.2 (50.6–58.1)	52.6 (49.6–58.5)	<0.001
RA min volume (mL)	35.3 (32.9–38.7)	34.2 (32.6–38.4)	<0.001

Note: Continuous variables are presented as median (IQR) unless otherwise stated. BMI, body mass index; SBP, systolic blood pressure; DBP, diastolic blood pressure; eGFR, estimated glomerular filtration rate; LA, left atrial; RA, right atrial; PA-TDI, the onset of the P wave in lead II and the peak A^′^ wave on tissue Doppler imaging; Ch, chamber; HDL, high-density lipoprotein; LDL, low-density lipoprotein; HbA1c, glycated hemoglobin.

**Table 2.** **Univariate correlates of change in mean PA-TDI duration vs. baseline and change in variables**.

Variable	Δ mean PA-TDI Duration vs. Baseline Variable		Δ mean PA-TDI duration vs. Δ variable	
	*R*	*p*	*R*	*p*
Age (years)	0.440	0.655		
BMI (kg/m^2^)	–0.069	0.480	0.094	0.044
SBP (mmHg)	–0.027	0.783	0.009	0.924
DBP (mmHg)	0.052	0.597	–0.044	0.656
eGFR (mL/min/1.73 m^2^)	0.119	0.221	–0.144	0.139
LDL cholesterol (mg/dL)	0.055	0.577	–0.006	0.951
HDL cholesterol (mg/dL)	0.037	0.705	0.031	0.751
HbA1c (%)	–0.109	0.264	0.163	0.096
Hematocrit (%)	–0.021	0.828	0.147	0.132
Lateral E/e′ ratio	0.025	0.805	0.312	0.001
Septal E/e′ ratio	0.075	0.451	0.249	0.010
E/A ratio	0.102	0.306	–0.382	<0.001
LA max volume	–0.023	0.812	0.038	0.694
LA min volume	0.034	0.730	0.019	0.843
RA max volume	0.019	0.846	0.099	0.313
RA min volume	0.043	0.657	0.031	0.747

**Table 3.** **Multiple linear regression analysis of predictors for changes in mean PA-TDI duration**.

Independent variables	Unstandardized β	Standard error	95% Confidence interval (CI)	*p*-value
Age (years)	0.031	0.074	[–0.115, 0.177]	0.679
Sex (male/female)	–1.498	1.358	[–4.182, 1.186]	0.272
Δ BMI (kg/m^2^)	0.454	0.201	[0.056, 0.852]	0.026
Lateral E/e′ ratio	0.110	0.032	[0.046, 0.174]	0.001

All delta (Δ) values were calculated as (value at six months after treatment – value at baseline)/value at baseline × 100 (%). A *p*-value < 0.05 was considered significant. We have revised the Δ value calculation as post-treatment minus baseline, consistent with standard statistical practice. Additionally, we evaluated multicollinearity using Variance Inflation Factors (VIF), and all VIF values were below 2.0, indicating no multicollinearity concerns. Formal power calculation was not performed because of the exploratory nature. Although no a priori sample size calculation was performed due to the exploratory design, a post hoc power analysis based on the observed reduction in mean PA-TDI (TACT) demonstrated adequate statistical power (approximately 95–98%) at a two-sided alpha level of 0.05.

## 3. Results

A total of 107 patients (57 female, 50 male) were included in the study; 57 patients were on dapagliflozin, and 50 were on empagliflozin. The mean age of the participants was 54 years (range: 49–62 years), and 42.1% had hypertension, while 15.9% had cardiovascular disease (CVD). The mean duration of SGLT2 inhibitor use was 183 days. The drugs administered prior to this study are shown in Table 4.

**Table 4.** **Baseline characteristics of the study population**.

Characteristic		All patients (n = 107)
Age (years), median (IQR)		54 (49–62)
Sex (female), n (%)		57 (53.3)
SGLT2i therapy duration (days)		183 (169–198)
Comorbidities, n (%)		
	Hypertension	45 (42.1)
	Cardiovascular disease (CVD)	17 (15.9)
	Smoking	31 (29.0)
Medications, n (%)		
	RAAS blockers	50 (46.7)
	Anti-aggregant agents	23 (21.5)
	Statin	17 (15.9)
	Fenofibrate	2 (1.9)
	Beta-blockers	21 (19.6)
	Calcium channel blockers	28 (26.2)
	Hydrochlorothiazide	14 (13.1)
	Spironolactone	3 (2.8)
Type of SGLT2i, n (%)		
	Empagliflozin	50 (46.7)
	Dapagliflozin	57 (53.3)

SGLT2i, sodium-glucose cotransporter 2 inhibitor; RAAS, renin-angiotensin-aldosterone system; IQR, interquartile ranges.

The clinical, anthropometric, laboratory and echocardiographic parameters at baseline and six months after starting treatment with SGLT2 inhibitors are shown in Table 1. BMI, systolic blood pressure, diastolic blood pressure, creatinine, total cholesterol, triglycerides, low-density lipoprotein (LDL) cholesterol, and glycated hemoglobin (HbA1c) level statistically decreased after six months of treatment (*p* < 0.001, *p* < 0.001, *p* < 0.001, *p* < 0.001, *p* = 0.011, *p* = 0.034, *p* = 0.005 and *p* < 0.001, respectively). With respect to the echocardiographic parameters, the PA-TDI septal, lateral, posterior, anterior, inferolateral, RA free wall and mean duration values as well as the lateral E/e^′^ and septal E/e^′^ ratios and RA max and min volumes statistically decreased (all *p <* 0.001). Further, the hematocrit and high-density lipoprotein (HDL) cholesterol values and E/A ratio statistically increased (*p* < 0.001, *p* = 0.007, *p* < 0.001, respectively). In an exploratory subgroup analysis, no significant difference in Δ TACT was observed between patients receiving dapagliflozin and empagliflozin (*p* > 0.05).

The univariate correlation analysis of changes in the mean PA-TDI duration showed that the mean PA-TDI was correlated with Δ BMI (r = 0.094, *p* = 0.044) and Δ lateral E/e^′^ ratio (r = 0.312, *p* = 0.001) (Table 2). Multiple regression analysis showed that Δ BMI and Δ lateral E/e^′^ ratio (*p* = 0.026 and *p* = 0.001, respectively) were inversely related to mean PA-TDI duration. These findings remained after adjusting the Δ mean PA-TDI value for age and sex (Table 3).

## 4. Discussion

The main finding of this prospective pilot study is that 6 months of sodium-glucose cotransporter 2 inhibitors (SGLT2i) treatment significantly shortens echocardiography-derived TACT in patients with T2DM and preserved left ventricular ejection fraction (Pre SGLT2i: 121.1 (111.6–129.8) ms, Post SGLT2i: 110.5 (101.1–119.6) ms, *p* < 0.001). The reduction in mean TACT values, as measured by PA-TDI time after six months of treatment, suggests that SGLT2 inhibitors may have potential positive effects on atrial structural and electrical remodeling. Although the reduction in TACT was statistically significant, the clinical relevance of an approximately 10 ms decrease remains uncertain. Since arrhythmic outcomes such as incident atrial fibrillation were not evaluated, no direct conclusions can be drawn regarding clinical benefit. Previous experimental and clinical studies have shown that DM causes atrial remodeling through atrial fibrosis, hypertrophy, and ion channel dysfunction [19, 20]. As a result of these processes, TACT is prolonged, and the risk of developing AF increases. TACT is a non-invasive and reproducible marker used to predict AF, reflecting atrial fibrosis and electrical heterogeneity [21]. In our study, the significant shortening of TACT after SGLT2 inhibitor treatment suggests that diabetes-related atrial remodeling may be at least partially reversible.

The cardiovascular benefits of SGLT2 inhibitors have been demonstrated in numerous large randomized clinical trials. Studies such as EMPA REG OUTCOME, CANVAS, and DECLARE TIMI 58 have shown that these agents reduce cardiovascular mortality and hospitalizations due to heart failure [2, 3, 5]. However, the underlying mechanisms of these benefits are not fully understood. Experimental studies have shown that SGLT2 inhibitors reduce oxidative stress, improve mitochondrial function, and suppress myocardial fibrosis [22]. This reduction in atrial fibrosis may be one of the possible mechanisms for the TACT shortening observed in our study. Supporting these events, a study by Soliman *et al*. [23] observed that the use of SGLT2i in diabetic patients undergoing catheter ablation for AF reduced the recurrence rate of AF. This observation suggests that SGLT2i treatment may have a significant effect on the electrophysiological balance of the atria [23].

Studies examining the effects of SGLT2 inhibitors on atrial arrhythmia burden are limited in the literature. Although no significant between-group difference was observed between dapagliflozin and empagliflozin in our study, the analysis was exploratory and the study was not specifically powered for a head-to-head comparison. A meta-analysis reported that SGLT2 inhibitors reduced the incidence of AF [24]. Similarly, an observational study reported a lower risk of new-onset AF in diabetic patients using empagliflozin [25]. However, these studies did not evaluate direct atrial electrical remodeling indicators such as atrial conduction times or TACT. In this respect, our study is one of the rare studies that demonstrates the effect of SGLT2 inhibitors on atrial conduction characteristics echocardiographically. It is noteworthy that in our study, the change in TACT was found to be independently and significantly associated with changes in BMI and lateral E/e^′^ ratio. This suggests that improvements in metabolic status and diastolic performance may contribute to the shortening of atrial conduction time. Obesity contributes to atrial enlargement and fibrosis through increased atrial pressures and inflammation, and is associated with the prolongation of TACT [26]. Weijs *et al*. [27] showed that the duration of PA-TDI is increased in the presence of high BMI. In our current study, we observed a significant decrease (*p* < 0.001) in BMI (kg/m^2^) with 6 months of SGLT2i treatment. In multiple regression analysis, we found that Δ BMI (95% CI: 0.056–0.852, *p*: 0.026) was an independent prognostic factor of mean PA-TDI. The use of SGLT2 inhibitors reduced the mean duration of PA-TDI. This may be due to a regression in diastolic dysfunction. In diabetic patients, an increase in the E/e^′^ ratio, an indicator of worsening diastolic function, is associated with the development of heart failure and increased mortality, independent of hypertension and coronary heart disease [28]. A study by Chao *et al*. [12] showed that the PA-TDI duration was significantly increased at different stages of diastolic dysfunction. The weight loss achieved by SGLT2 inhibitors may have contributed to the shortening of conduction time by reducing atrial load. Matsutani *et al*. [15] found that after three months of treatment with SGLT2i (canagliflozin), patients with T2DM could improve ventricular diastolic function (E/e^′^). In parallel with this study, our research observed a decrease in septal [pre-SGLT2i: 8.3 (7.1–10), post-SGLT2i: 7.5 (6.2–8.8), *p* < 0.001] and lateral [pre-SGLT2i: 7 (5.8–8.8), post-SGLT2i: 6 (5–7.5), *p* < 0.001] E/e^′^ ratios after approximately 183 days of SGLT2i treatment. This may reflect an improvement in diastolic function and a decrease in left atrial pressure. Diastolic dysfunction is known to be associated with atrial remodeling and AF development [29]. In conclusion, we believe that the hemodynamic and metabolic effects of SGLT2 inhibitors have positive indirect contributions to atrial conduction.

The reversibility of TACT has been addressed to a limited extent in the literature. There are few studies reporting a reduction in TACT after hypertension treatment or weight loss [29, 30]. The results of our study are important because they show that TACT can be significantly reduced with a pharmacological agent, moreover, with a class of drugs used primarily for glycemic control. Considering all factors, the reduction in TACT observed in our study should not be interpreted as a direct pharmacological effect of SGLT2 inhibition alone. Given the concurrent improvements in BMI, glycemic control, blood pressure, lipid profile, and diastolic parameters, the shortening of atrial conduction time may predominantly reflect improved hemodynamic loading conditions and metabolic status rather than a direct anti-arrhythmic effect or reduction in atrial fibrillation risk.

This prospective pilot study demonstrated that SGLT2 inhibitor therapy significantly reduced total atrial conduction time in patients with T2DM. This finding suggests that the cardiovascular benefits of SGLT2 inhibitors may not be limited to ventricular function but may also encompass atrial electrical and structural remodeling. Randomized controlled trials with long-term follow-up in larger patient populations are necessary to confirm these effects and demonstrate their clinical significance.

### Limitations

The absence of a control group makes it impossible to definitively determine whether possible improvements are due to SGLT2 inhibitors or other factors; therefore, the lack of a control group is a significant limitation. The relatively small number of participants is a significant limitation; multicenter studies with more participants are needed. The follow-up period was relatively short. The long-term effects of SGLT2i need to be observed. The study investigated two different molecules: dapagliflozin and empagliflozin. It is unclear whether this is a class effect or a direct effect of the molecules themselves. The echocardiographic measurement of TACT is operator-dependent, and although performed by two different cardiologists, this operator dependence is another limitation. Residual confounding remains possible because not all potential clinical and metabolic covariates could be included in the final regression model. The shortening of TACT time observed with SGLT2i use was correlated, not causal.

## 5. Conclusions

In this study, SGLT2 inhibitor therapy was associated with a decrease in TACT in patients with type 2 diabetes. This finding should be considered hypothesis-generating and may reflect improvements in diastolic parameters, atrial electromechanical properties, and cardiometabolic status.

## Data Availability

The datasets used and analyzed during the current study are available from the corresponding authors upon reasonable request.

## Abbreviations

SGLT2i, sodium-glucose cotransporter 2 inhibitors; T2DM, type 2 diabetes mellitus; TACT, total atrial conduction time; PA-TDI, the onset of the P wave in lead II and the peak A^′^ wave on tissue Doppler imaging; HbA1c, glycated hemoglobin; BMI, body mass index; AF, atrial fibrillation; EFs, ejection fractions; MDRD, Modification of Diet in Renal Disease; MINOCA, myocardial infarction with nonobstructive coronary arteries; PCI, percutaneous coronary intervention; ECGs, electrocardiograms; SBP, systolic blood pressure; DBP, diastolic blood pressure; GFR, glomerular filtration rate; eGFR, estimated glomerular filtration rate; LA, left atrial; RA, right atrial; ASE, American Society of Echocardiography; EACVI, European Association of Cardiovascular Imaging; IQR, interquartile range; CVD, cardiovascular disease; Ch, chamber; HDL, high-density lipoprotein; LDL, low-density lipoprotein.
